# Breast Cancer Knowledge and Preventive Practice Among Graduate Students: A Scoping Review

**DOI:** 10.3390/cancers18071147

**Published:** 2026-04-02

**Authors:** Binita Adhikari, Xan Goodman, Md Maksudul Alam, Miguel Antonio Fudolig, Gabriela Buccini, Nicole V. DeVille

**Affiliations:** 1 Department of Epidemiology and Biostatistics, School of Public Health, University of Nevada, Las Vegas, NV 89119, USA; 2 Research and Education, UNLV University Libraries, University of Nevada, Las Vegas, NV 89119, USA; 3 Department of Social and Behavioral Health, School of Public Health, University of Nevada, Las Vegas, NV 89119, USA

**Keywords:** breast cancer, knowledge, cancer prevention, screening

## Abstract

Background/Objectives: Breast cancer is one of the most prevalent cancers among women, with notable increases among women younger than 50 years. Knowledge about breast cancer and preventive measures (e.g., early detection) are key to reducing breast cancer morbidity and mortality. Many graduate students fall within an age range when breast cancer risk starts to rise. However, research focused specifically on graduate students’ breast cancer knowledge and practices of preventive measures are sparse. Methods: This scoping review aims to synthesize the literature on breast cancer knowledge and practice of preventive measures among graduate students in a global context. Four databases (PubMed, CINAHL, APA PsycINFO, Embase) were searched for articles published between 2014 and 2024, and the following inclusion criteria were applied: full-text peer-reviewed articles available online; target population includes graduate students aged 20 to 50 years; English language; and, cross-sectional, cohort, case–control, ecological, and experimental/intervention studies. Two reviewers independently conducted article screening and data extraction using Covidence. Results: Sixteen studies met the selection criteria. Knowledge of breast cancer was examined in 94% (15/16) of the included studies and 75% (12/16) of the studies assessed practice of prevention measures (e.g., breast self-examination) among graduate students. Overall, most studies reported poor knowledge and limited uptake of prevention practices. Educational background (e.g., years of education, academic discipline and GPA), access to healthcare services, and other socioeconomic characteristics were commonly reported factors significantly associated with breast cancer knowledge and practice of preventive measures in graduate students. Conclusions: These findings may inform targeted educational interventions to increase knowledge and promote the early detection and prevention of breast cancer among graduate students.

## Simple Summary

Breast cancer is both preventable and highly treatable when detected early, making awareness of preventive measures critical. This scoping review synthesized 16 studies examining graduate students’ knowledge of breast cancer, including risk factors, signs and symptoms, and recommended screening methods. While a growing body of research exists, most studies have focused on students in health-related fields, with limited attention paid to those in non-health disciplines. Overall, the findings indicate that graduate students demonstrate insufficient knowledge and low engagement in preventive practices. These gaps underscore the need for targeted health education interventions within university settings to improve breast cancer awareness and promote preventive behaviors among graduate students.

## 1. Introduction

Breast cancer is a disease in which breast cells grow out of control, resulting in tumors [1]. Globally, breast cancer is one of the most prevalent cancers [2] and is the most common cancer among women [3,4]. The burden of breast cancer is increasing worldwide in developing and developed countries [2]. One in eight women will be diagnosed with breast cancer in their lifetime [5]. In the United States, breast cancer accounts for almost 15% of all new cancer cases and 7% of cancer-related deaths [6]. Early detection through breast cancer screening and awareness of risk factors is key to reducing morbidity and mortality from breast cancer and improving survival outcomes.

The most common breast cancer screening methods for early detection are mammograms, breast magnetic resonance imaging (MRI), clinical breast examination (CBE), and breast self-examination (BSE) [7]. Mammograms are X-rays that can detect asymptomatic breast cancer and are completed by healthcare providers. Breast MRI is recommended along with a mammogram to screen women who are at higher risk of developing breast cancer [7]. A CBE is performed by a doctor or a nurse who feels for lumps or other changes in the breast [7]. BSE is a simple, at-home method that involves regularly examining and feeling the breasts in front of a mirror to look for any lumps or changes [7]. Screening methods like mammography for early detection of breast cancer can reduce breast cancer mortality by 20% [8,9].

Previous studies have shown that several factors are associated with breast cancer screening behavior [10,11]. Sociodemographic characteristics including age, educational attainment, ethnicity, marital status income, and healthcare access have been associated with breast cancer screening practice [12,13]. Further, awareness of breast cancer, health beliefs (e.g., perceived risk of breast cancer screening methods), and healthcare providers’ screening recommendations are related to screening behavior [13,14,15]. Most importantly, health knowledge is a crucial factor predicting health behavior; thus, one’s knowledge about breast cancer may affect prevention practices, such as participation in breast cancer screening methods [16,17].

While knowledge of breast cancer has been associated with participation in preventive practice [18,19], gaps in general knowledge of breast cancer risk factors, signs and symptoms, and screening practices persist, even among educated women; these gaps highlight the need for improved and targeted breast cancer education and interventions [20]. Prior studies have focused on exploring knowledge of breast cancer signs, symptoms, and screening methods among the general population, women who are at risk of developing breast cancer (i.e., those aged 40 years or older), and women who have positive family history of breast cancers [21] and are genetically at risk [22]. However, recent trends of breast cancer in the global context suggest that incidence is also increasing in younger women aged 20 to 49 years [23,24]. While graduate students’ higher educational attainment may suggest a greater likelihood of engaging in health-seeking behaviors [14], graduate students often face barriers to accessing healthcare services. Many graduate students are non-traditional learners who balance academic responsibilities with employment, family care, and financial insecurity [25,26]. Research shows that graduate students frequently experience high levels of stress, time constraints, and limited income, which can impede routine healthcare access, including preventive services, such as mammograms or clinical breast exams [24,26,27].

In many cases, graduate students are underpaid or uninsured, and even when covered by student health plans, those plans may not include or emphasize preventive services like cancer screenings [28,29]. Further, graduate students may demonstrate established correlates of breast cancer risk, including lower physical activity, nulliparity, and delayed age at first birth [30]. Despite many graduate students falling within the age range when breast cancer risk begins to rise, their health behaviors remain underexplored in breast cancer research. Using a scoping review approach, we aim to synthesize the literature on breast cancer knowledge and preventive practices among graduate students globally. Specifically, the research questions for this study are as follows:

1. Does knowledge about breast cancer risk factors, signs and symptoms, and screening methods differ based on graduate students’ sociodemographic characteristics and academic discipline?
2. How prevalent are breast cancer preventive practices (e.g., screening) among graduate students globally?

## 2. Materials and Methods

A scoping review approach was used, which is appropriate for mapping key concepts and identifying gaps in the literature [31]. The review followed Joanna Briggs Institute (JBI) methodological guidelines [32], and reporting adhered to the 2020 PRISMA Extension for Scoping Reviews [31]. The protocol was not previously registered.

### 2.1. Search Strategy

The research question was guided by the PCC format for scoping reviews, where P stands for population, C for concept, and C for context [33]. In this review, P (population) is graduate students, C (concept) is knowledge and preventive practice of breast cancer, and C (context) is university and college settings. The four databases used for the literature search were PubMed, CINAHL, APA PsycINFO, and Embase. Databases were searched using keywords and MESH terms generated with a Health Science librarian (XG): “Breast cancer” [Mesh] AND (“cancer screening” [ot] OR “early detection of cancer” [ot] OR “breast self-examination” [Mesh] OR screening [ot] OR “screening practices” [ot] OR practices [ot] OR prevention [ot]) AND (knowledge OR awareness) AND (“university students” OR “graduate students” OR “college student” OR “medical students OR students, health occupations” [Mesh]). The review included studies published between 2014 and 2024 to capture recent evidence in breast cancer research on knowledge, risk perception, and preventive behaviors in light of advances in screening guidelines and ongoing public health initiatives. The searches were conducted on 11 February 2025, and the search strings used for PubMed are provided as an example in Table S1.

### 2.2. Eligibility Criteria and Study Selection

We applied the following inclusion criteria: (1) full-text peer-reviewed journal articles available online; (2) publication date between 2014 and 2024; (3) target population includes graduate students aged 20 to 50 years; (4) articles written in English language; and (5) cross-sectional designs, cohort studies, case–control studies, ecological studies, and experimental/intervention studies. Case reports, case series, conference proceedings, commentaries, editorials, summaries, animal studies, and articles which were only published as dissertations, posters, abstracts, or presentations were excluded.

All titles and abstracts were independently screened by two reviewers (BA and MA), with disagreements resolved by a third reviewer (NVD). Full-text articles were independently reviewed for inclusion eligibility by two coauthors (BA and MA), with substantial inter-rater reliability (Cohen’s kappa = 0.76). Any disagreements were resolved in consensus with an additional coauthor (NVD).

### 2.3. Data Extraction

We used a modified version of Covidence’s (Covidence, Melbourne, Australia) standardized data extraction template (version 2.0) to extract data from the studies deemed eligible for inclusion in accordance with the 2020 PRISMA Extension for Scoping Review guidelines [31]. Data extracted from articles that met selection criteria included study ID, author, publication year, setting, study design, research objective, population, overall knowledge of breast cancer, proportion of participants with knowledge of breast cancer risk factors, proportion of participants with knowledge of signs and symptoms, and proportion of participants with knowledge of breast cancer screening methods (Table 1). Data extraction items are defined in a codebook, shown in Table S2. Data extraction was conducted by two authors independently for each study (BA, MA), and disagreements were resolved by consensus.

### 2.4. Variables of Interest

#### 2.4.1. Factors Examined Across Studies

In this scoping review, we extracted variables that were examined in relation to breast cancer knowledge and preventative practices among graduate students, as reported in the included studies. Consistent with scoping review methodology, these variables were not treated as fixed independent or dependent variables, but rather as factors whose roles varied depending upon the study design and analytic approach.

The most commonly examined factors included sociodemographic and academic characteristics such as age, marital status, race/ethnicity, level of education, academic discipline, years of study, grade point average (GPA), family history of breast cancer, and socioeconomic indicators (e.g., income). Several studies also examined healthcare access-related factors in relation to breast cancer knowledge and preventive practices.

#### 2.4.2. Outcomes of Interest

The primary outcome of interest of this review was breast cancer knowledge among graduate students. Broadly defined, knowledge and understanding of breast cancer involves a comprehensive awareness of both modifiable and non-modifiable factors that increase risk, identification of signs and symptoms, and awareness and adoption of prevention methods to minimize risk or support early detection [2]. Knowledge was measured using author-developed or previously validated questionnaires and reported as composite scores, categorical levels (e.g., poor, moderate, good), or proportions of correct responses to individual items. The secondary outcome was adoption of breast cancer preventive practices, including engagement in breast self-examination (BSE), clinical breast examination (CBE), and mammography screening. Preventive practices were generally measured through self-reported behaviors, including whether participants had ever performed breast self-examination (BSE), how often they practiced BSE, and whether they had previously participated in clinical screening.

#### 2.4.3. Classification of Breast Cancer Knowledge Levels

Across the included studies, breast cancer knowledge was examined either as an outcome variable or as an explanatory factor associated with preventive practices. Knowledge was measured using heterogenous instruments, scoring systems, and classification thresholds. Some studies categorized knowledge levels using labels such as poor, moderate, or good, while others reported continuous scores or item-level responses. In this scoping review, all classifications of breast cancer knowledge (e.g., poor, moderate, or good knowledge) were extracted and summarized exactly as defined by the original study authors, without reclassification or standardization; measurement and reporting approaches are detailed in Table 2.

### 2.5. Data Synthesis

First, study characteristics were summarized, followed by a thematic analysis of the data items extracted. One co-author (BA) used deductive content analysis to organize the extracted data into themes [50], including overall knowledge of breast cancer, knowledge of BC risk factors, knowledge of signs and symptoms, knowledge of breast cancer screening methods, knowledge of preventive practices, factors influencing knowledge and preventive practices, gaps between knowledge and practice, and barriers to breast cancer screening practice. Additional co-authors (MA, NVD) independently reviewed and verified coding to enhance the rigor of the thematic analysis.

## 3. Results

### 3.1. Study Characteristics

**Figure 1:**
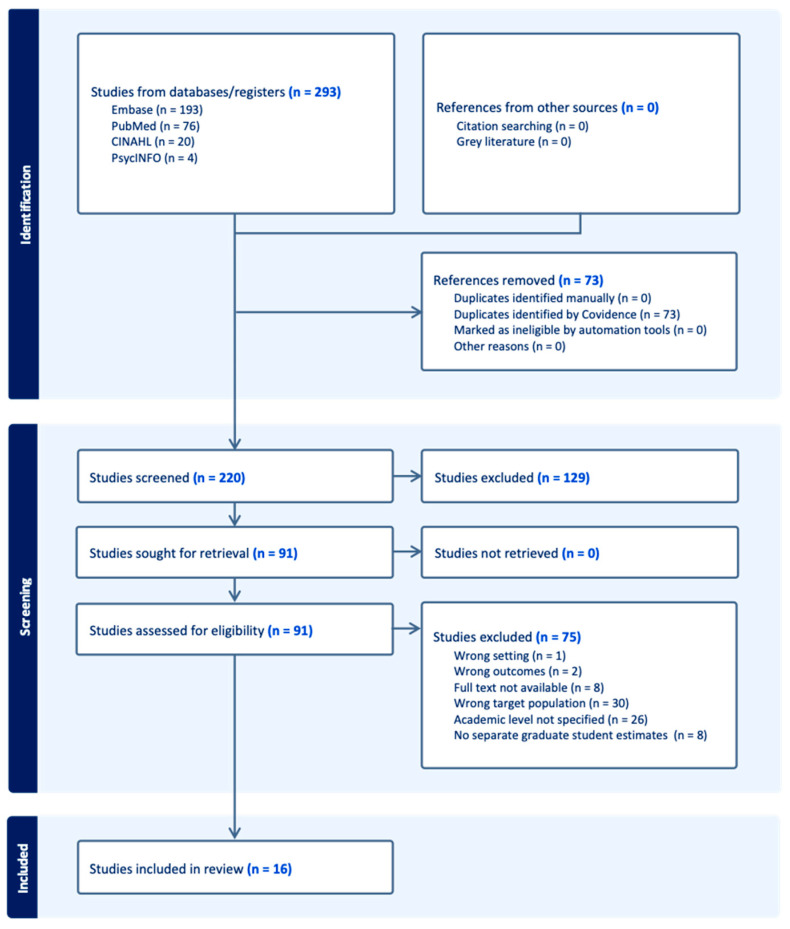
PRISMA diagram documenting the selection and screening process for included studies from four databases.

We followed PRISMA scoping review guidelines (Table S3) [31], and the full article selection process is documented in a PRISMA flow chart (Figure 1). A total of 293 records were identified through database searches, with no additional records identified through other sources. After removing duplicate records (*n* = 73), 220 title and abstracts were screened, of which 129 records were excluded. A total of 91 full-text articles were assessed, with 75 excluded due to wrong setting (*n* = 1), wrong outcomes (*n* = 2), unavailable full text (*n* = 8), wrong target population (*n* = 30), unspecified academic level (*n* = 26), or lack of separate graduate student estimates (*n* = 8).

Sixteen studies met the selection criteria (Table 1) [34,35,36,37,38,39,40,41,42,43,44,45,46,47,48,49]. Study locations included Malaysia (*n* = 3), Bangladesh (*n* = 3), India (*n* = 3), United States (*n* = 1), Lebanon (*n* = 1), Iran (*n* = 1), China (*n* = 1), Sudan (*n* = 1), Egypt (*n* = 1), and Pakistan (*n* = 1) (Figure S1).

Most of the studies (*n* = 15) were cross-sectional, and one was an intervention study (Table 1). A majority of the studies aimed to assess knowledge, attitudes, and practices (KAP) related to breast cancer awareness, screening, and breast self-examination (BSE). Among the 16 included studies, 15 studies assessed the overall knowledge of breast cancer [34,35,36,37,38,39,40,41,42,44,45,46,47,48,49] and 12 studies evaluated graduate students’ breast cancer screening practices [34,35,36,38,39,41,43,44,45,46,47,49].

### 3.2. Knowledge of Breast Cancer

The included studies reported knowledge across various dimensions of breast cancer. In these studies, “knowledge” was defined by the authors based on the specific questionnaires or other tools used to assess participants’ understanding of breast cancer risk factors, signs and symptoms, and preventive practices, as detailed in Table 2. Knowledge regarding breast cancer was reported by authors of the included studies as “poor”, “moderate”, and “good” using total knowledge scores in some studies. Other studies categorized knowledge score by reporting prevalence [34,40,42,46].

Most of the studies demonstrated that the overall knowledge of breast cancer among graduate students in the global context was poor (Table 2). In this review, only 12% of the studies (*n* = 2) indicated a “good” level of knowledge about breast cancer among graduate students [36,47]. More than half of the studies (*n* = 10) showed “poor” breast cancer knowledge among graduate students [34,35,37,40,41,42,45,46,48,49], three studies indicated “moderate” knowledge among graduate students [38,39,44], and one study did not measure level of knowledge [43].

#### 3.2.1. Knowledge of Breast Cancer Risk Factors

Knowledge of breast cancer risk factors was one dimension measured by researchers. Of the 16 studies, knowledge about breast cancer risk factors was assessed in 10 studies [34,35,36,41,42,44,45,46,47], and 6 of the studies did not report risk factor knowledge among graduate students [38,39,40,43,48,49]. Out of the ten studies reporting knowledge of risk factors, six studies reported “poor” understanding of those risk factors among graduate students [34,35,36,37,42,45]. For example, a cross-sectional study conducted in India by Das et al. (2022) found that only 36.4% of graduate students correctly identified different BC risk factors [34]. Similarly, a study from Lebanon by Deeb et al. (2024) measured the level of knowledge related to lifestyle risk factors (e.g., nutrition) and was the only study that examined both modifiable and nonmodifiable risk factors [35]. The authors found that students had poor knowledge regarding nutrition-related factors (e.g., red meat consumption, alcohol consumption, fruit and vegetable consumption) associated with breast cancer risk. For instance, almost two thirds of the students had poor knowledge regarding nutrition-related risk factors of BC [35]. In contrast, those students who had an extensive knowledge of risk factors of breast cancer were medical students and other students enrolled in health-related majors [41,47]. A cross-sectional study conducted among medical students in Iran found that the proportion of the students who correctly identified breast cancer risk factors (e.g., aging, family history, chest radiation therapy) ranged from 60 to 80% [41]. Similarly, another study among medical students in India found that more than 80% of the medical students surveyed were aware of breast cancer risk factors [47]. Additionally, an intervention study conducted in Bangladesh among female students revealed that the mean score of knowledge about breast cancer risk factors increased after an educational intervention highlighting the effectiveness of health education programs to increase knowledge and promote prevention practices [45].

#### 3.2.2. Knowledge of Breast Cancer Signs and Symptoms

Almost half (*n* = 8) of the included studies measured knowledge about breast cancer signs and symptoms [34,37,39,42,44,45,46,47]. Six studies indicated that students had “poor” understanding of the signs and symptoms of breast cancer [34,37,42,44,45,46]. One study done among graduate female students in India reported that only 42.02% of graduate students had knowledge of one or more BC sign and symptom [34]. Likewise, a national survey in Pakistan by Hussain et al. (2022) indicated that the majority of graduate students in their study did not know major breast cancer signs and symptoms [37]. Those who had a “good” level of understanding about breast cancer signs and symptoms were enrolled in a medical graduate program [39,47]. Satapathy et al. (2022) conducted a study among medical students enrolled in graduate and postgraduate degrees, and all knew that presence of skin irritation and dimpling are symptoms of breast cancer [47]. Another study reported that although most students (88.75%) had heard about breast cancer only 60.62% of them were aware of its signs and symptoms [43]. A cross-sectional study conducted in Pakistan found that students enrolled in master’s programs had higher mean scores of knowledge about breast cancer signs and symptoms as compared to students that were enrolled in undergraduate programs [46].

#### 3.2.3. Knowledge of Breast Cancer Screening Methods

Eleven studies reported on the knowledge of breast cancer screening methods [34,36,38,39,40,42,45,46,47,48]. The reviewed studies indicated knowledge of the most common screening methods for early detection of breast cancer (e.g., BSE, mammogram and CBE). Out of eleven studies that reported about knowledge of screening methods, nine of them indicated students had poor knowledge [34,37,38,40,42,46,47,48]. Most of the students had heard about BSE [39,42,47]. Most of the students recognized the importance of screening methods for detecting breast cancer at an early stage. For instance, Haque et al. (2016) indicated that 91.1% of the students in health-related majors acknowledged the importance of regular mammograms and BSE [37]. Though students were aware of BSE, they demonstrated inadequate knowledge regarding how to perform BSE, the recommended frequency of practicing BSE, and the approximate age to start BSE [37,40,41,43]. Prachishree et al. (2023) reported that only 36% of the students knew how to perform BSE [42]. Hussain et al. reported that only 25.1% of graduate students knew the best time for self-examination is one week after the onset of menstruation, and only one fourth of the students correctly stated that BSE should be performed monthly [37]. Knowledge regarding CBE and mammograms was explored in only a few of the studies [34,36,47]. These studies showed that students knew about the importance of mammograms and CBE to detect breast cancer very early but only a few students knew that mammography should be initiated after the age of 40 years [36,37,42,47]. Medical students were aware of other screening methods like ultrasound, MRI, and CT scan [40,47].

### 3.3. Preventive Practices of Breast Cancer Among Students

Preventive behaviors like practice of BSE were explored in ten studies [34,35,36,38,39,41,43,44,47,49], and three studies examined practices of CBE and mammogram among students [36,37,47]. However, the prevalence of BSE practice varied across the studies (range: 29–75%). In the study by Haque et al. (2022), only 36% of participants reported performing BSE once a year [36], whereas Das et al. found that only 29.8% of the graduate students had ever performed BSE [34]. Ishtiak et al. (2022) found that only 10.7% of participants who were aware of BSE practiced on a monthly basis [38]. The regular practice of BSE was low even among medical students. For instance, in one study done among medical graduate students, BSE was ever practiced by 75.61% of medical students; however, only 23.17% of those students performed BSE monthly [47].

Among those studies that reported the practices of other screening methods like CBE and mammography, the uptake of such screening methods was low [34,36,47]. For example, Satapathy et al. found that only 9% of respondents had ever undergone a CBE, while none reported having a mammogram, primarily due to their young age [47]. Mammograms are recommended starting at the age of 40 for those at average risk [47]. Haque et al. found that 76.8% of the students had “good” knowledge about mammograms and recognized their importance, but only 2% of those students underwent mammograms [36]. The majority of the studies’ participants were younger than the recommended age for mammogram screening.

Overall, the adoption of preventive practice related to breast cancer among students was mixed, with some engaging in BSE irregularly and few accessing clinical screening services (e.g., CBEs and mammograms) [34,38,39,41,47].

### 3.4. Factors Associated with Knowledge of Breast Cancer and Preventive Practices

Across the studies included, several sociodemographic and academic factors were significantly associated with knowledge of breast cancer (Table 3). Age was one of the most frequently reported significant factors influencing knowledge of breast cancer risk factors, signs and symptoms and recommended screening methods [34,36,38,45]. Academic-related variables such as major of study, GPA, years of education, and academic level were also significantly associated with higher knowledge levels of breast cancer [35,37,38,43,44,46]. In contrast, factors like marital status, residence, and parents’ educational level were often reported as not significantly associated with knowledge, highlighting some inconsistencies across populations (Table 3).

Preventive practices, particularly breast self-examination (BSE), were associated with a different set of factors. Higher education level, a family history of breast cancer, and greater knowledge scores were consistently linked to increased engagement in preventive behaviors. However, some variables, including age, marital status, and type of university (private vs. public), showed inconsistent or non-significant associations with preventive practices across studies (Table 3).

An intervention study conducted among female university students revealed significant changes in knowledge and awareness about breast cancer and BSE practices after an educational intervention [45]. Health education interventions, including group-based health educations sessions delivered in small groups of 10–15 students through interactive lectures/discussion and brainstorming with practical demonstration of BSE using leaflets and take-home materials, were conducted for fifteen days; pre- and post-intervention knowledge scores (regarding risk factors, symptoms, and screening methods) were significantly higher among students who received the education intervention. Additionally, BSE practice among students increased from 21.3% (pre-intervention) to 33.8% (post-intervention) (*p* < 0.01) [45]. Evidence from this study highlights the effectiveness of educational interventions in improving the preventative behaviors among students.

### 3.5. Gap Between Knowledge and Practice

Several studies have documented a distinct gap between knowledge of breast cancer and practice of screening methods among students. Although awareness of BSE and mammography was generally “moderate” to “high” in some contexts, the uptake of these practices remained low [36,38,47]. For example, a cross-sectional study in Malaysia reported that 100% of the students knew about BSE and mammography and acknowledged both as important screening methods to detect cancer; however, only 2% of the students underwent mammograms [36]. Similarly, while 69% of students knew how to perform BSE, only 23% of the students reported performing BSE at least once a year [36].

Comparable findings were observed in another study conducted among female university students in Bangladesh. Although 60.5% had heard about BSE, approximately 80% of students who had adequate knowledge about BSE did not practice BSE, clearly demonstrating the gap between knowledge and practice [38]. The authors noted that nearly four fifths of medical students who had knowledge of BSE never practiced it, suggesting a clear gap between knowledge and practices in this population [38].

### 3.6. Barriers to Screening Practices Among Students

Several studies identified key barriers to the practices of breast cancer screening methods (BSE, CBE and mammogram) among students [34,36,37]. The most common reported personal barrier to practicing BSE and seeking healthcare related to breast cancer was “lack of time” [36,37]. Students reported barriers to accessing recommended screening services, including financial constraints that limited their ability to undergo mammography or clinical breast examinations, even when advised by medical personnel [36].

Emotional barriers were also identified in some studies. Some of the students shared that the main barrier to seeking healthcare was embarrassment [34], and other students admitted they were scared to see a doctor [34]. Some of the students also reported that they felt uncomfortable discussing symptoms with a healthcare provider [37]. Similarly, other barriers related to BSE practice reported across the studies were inadequate knowledge about BSE, which included not knowing the recommended age to start BSE, lack of clarity on the recommended frequency, and low confidence in correctly performing the technique [42,46,47]. Even though students had heard about BSE, they did not feel comfortable and confident enough to practice and stated that they lacked the information to perform BSE.

Barriers to mammography uptake among students included misconceptions and physician preference. In one study, researchers found that students had misconceptions about harmful radiation exposure during mammograms and pain during the procedure [42]. Another study reported that students preferred mammograms to be conducted by female doctors [37]. This preference may be due to cultural norms or religious beliefs, especially in certain communities where physical examination by a male provider is uncomfortable or unacceptable.

## 4. Discussion

This review aimed to examine knowledge about breast cancer and its risk factors, signs and symptoms, and screening methods (BSE, CBE and mammogram) and to assess the preventive practices of screening methods among graduate students globally. The included studies focused on graduate students from different fields of study, with several studies focused specifically on health-related disciplines [40,47,48]. The age of the students ranged from 20 years to 60 years. BSE was the most commonly reported preventive practice. The limited assessment of mammography and CBE practices among graduate students may be attributed to their younger age, as most do not fall within the recommended age range for routine mammography screening. The included studies presented mixed findings regarding graduate students’ knowledge of BC risk factors, signs and symptoms, and screening methods.

In most of the included studies (*n* = 10), knowledge of BC risk factors among graduate students was reported as poor, with correct identification of risk factors ranging from 36% to 90%. Several studies highlighted gaps in understanding modifiable lifestyle-related risks (e.g., diet and alcohol consumption), as well as incomplete recognition of established risk factors [35,36]. These findings are consistent with previous research demonstrating inadequate awareness of BC risk factors among students [12,51].

Knowledge of BC signs and symptoms among graduate students was similarly limited, ranging from 24% to 97%. These knowledge gaps are concerning because early recognition of breast cancer symptoms is critical for timely diagnosis and treatment, and delays in symptom identification are associated with poorer clinical outcomes [52]. Therefore, increasing awareness through targeted educational campaigns and training programs for graduate students across academic disciplines is essential to promote earlier detection and improve health outcomes.

This review revealed that while many students had heard of BSE, few possessed adequate knowledge of how and when to perform screening. This emphasizes the need for targeted health education that teaches and empowers students to perform BSE. Furthermore, only a small portion of students understood age-appropriate screening guidelines, such as the recommendation to begin mammograms at age 40 [40]. Medical students demonstrated higher awareness of advanced screening methods (e.g., MRI and ultrasound), but the overall knowledge gap among non-medical students remains a concern. These findings suggest the importance of targeted educational efforts that not only raise awareness of screening tools but also provide practical, age-appropriate guidance for practice.

As anticipated, the level of knowledge regarding different dimensions of breast cancer was higher in medical students and students in health-related majors compared to non-health-related major students. Previous studies have also found similar results indicating higher levels of breast cancer knowledge in health-related and medical students compared to non-health-related major students [53,54]. Most of the existing studies were conducted among students in health-related majors, emphasizing the need for more research in non-health-related major students. Three studies found that students from medical or health science disciplines had significantly higher awareness of BC and preventive practice rates than their non-medical counterparts [34,43,49].

Several studies included in this review identified significant factors associated with knowledge and preventive practice of breast cancer. For instance, most studies highlighted the influence of educational level on knowledge regarding breast cancer. Education level was a significant predictor of BC knowledge among students in several included studies. Students enrolled in graduate programs (e.g., master’s, doctorate) were more likely to have a better understanding of the risk factors, signs and symptoms and recognized the importance of screening methods for early detection of breast cancer [34,35,37,39,48,49]. Factors like health-related academic background (e.g., taking a public health or biology course, although not a health major), previous participation in breast cancer awareness campaigns, and exposure to breast cancer through family or peers were significantly associated with increased knowledge and higher rates of BSE practice. These variations underscore the importance of sociodemographic factors in shaping both knowledge and behavior related to breast cancer prevention.

Despite the recognized benefits of screening methods, several barriers to breast cancer prevention practices were identified among graduate students, including financial constraints, emotional, religious and social preferences, and lack of knowledge. Interpreting these barriers through the lens of the Health Belief Model provides a clear understanding of the factors that may influence a student’s willingness to engage in preventive behaviors. The Health Belief Model explains that individuals’ health behaviors are influenced by their perceptions of susceptibility, severity, benefits, barriers, and cues to action [55]. The perceived barriers by graduate students likely outweighed perceived susceptibility and perceived benefits of early detection, resulting in decreased motivation to engage in breast cancer preventive practices. Longitudinal research and interventions addressing these identified barriers, as well as providing cues to action (e.g., mobile app-based screening reminder system), are needed.

There is a persistent gap between breast cancer knowledge and engagement in preventive practice. While the younger age distribution of graduate students limits eligibility for routine mammogram screening, the low uptake of other preventive behaviors (e.g., BSE) is not justified. Further, students in non-health-related disciplines demonstrated poorer knowledge and preventive practice compared to students in health-related disciplines. Universities can take an active role and should prioritize addressing these gaps by integrating breast cancer information sessions and training (e.g., BSE demonstrations) into orientation sessions, health fairs, or other campus wellness initiatives. Additionally, implementing opt-in reminder systems for students that send personalized reminders via email, text/SMS, or social media platforms may support sustained practice of preventive behaviors [56].

To better understand the documented disconnect between knowledge and practice, it is necessary to consider the broader contextual, cultural, and structural factors that influence preventive behaviors, including demographic characteristics, sociocultural norms, and access-related constraints. In Malaysia, Haque and colleagues found that the predominantly Muslim study population expressed a conservative attitude toward mammography, with approximately one-third of participants requesting that the examination be conducted exclusively by a female physician [36]. Such a preference, while medically reasonable, may result in logistical barriers in contexts where female physicians may be scarce. Additionally, trust in spiritual healing practices and traditional therapies over biomedical interventions was documented among Pakistani students; women reported seeking formal healthcare only at the most advanced stages of disease, partly due to faith-based fatalism and reliance on non-clinical remedies [37]. From a socioeconomic standpoint, findings from the studies, especially those conducted in lower- and middle-income countries, reiterate an important point: mammography and advanced imaging remain financially inaccessible to large portions of the population. For example, Pakistan’s designation as a middle-income country, where nearly a quarter of the population lives below the national poverty line, means that the out-of-pocket costs associated with clinical breast examination and mammographic screening are prohibitive for many. This socioeconomic vulnerability reinforces the significance of cost-free, self-administered methods like BSE, particularly in under-resourced settings with accessibility issues.

In summary, this review underscores the need for targeted educational interventions that address not only knowledge deficits but also behavioral and systemic barriers to breast cancer prevention among graduate students globally. Only one study included in the review implemented an intervention in this population. The intervention study demonstrated a statistically significant increase in BSE practice, suggesting that integrating breast health education into university curriculum, coupled with practical demonstrations and peer-led awareness activities, may both enhance knowledge and motivate students to practice. Overall, the findings reveal a persistent gap between knowledge and practice, even among those with higher educational attainment, emphasizing the importance of tailored, practice-focused interventions.

### 4.1. Strengths

There are a limited number of studies focused on this topic among graduate students in the global arena. We captured some of the important dimensions of breast cancer knowledge among students, and our findings revealed some directions to develop evidence-based, targeted interventions that should be implemented in the university and college settings to promote prevention and early diagnosis/treatment and reduce the growing burden of breast cancer. We followed JBI guidelines for this scoping review, ensuring a transparent approach to study selection, data extraction, and synthesis. We collaborated with a health sciences research librarian during the development of the search strategy, enhancing the precision of our search strings and reducing the risk of missing relevant studies. Two independent reviewers conducted screening and data extraction and conflicts were resolved by a third reviewer, which minimized the bias and enhanced the reliability of the findings.

### 4.2. Limitations

Our scoping review has several limitations. Some selection bias may be present due to the databases selected and selection criteria imposed. In this study, we excluded those articles in which the academic level of the student was not clearly specified. While reviewing studies in different countries, we encountered variability in how the academic programs and academic level of the enrolled students were classified across countries and regions. For instance, studies with international medical students were excluded when it was unclear whether the students were enrolled in undergraduate or graduate programs (*n* = 26). Some publication bias may be present, as this review included published peer-reviewed results. This scoping review was conducted to map the existing evidence of knowledge of breast cancer and preventive practices among students and is not meant to be a comprehensive analysis of the topic. Findings are not generalizable to all women since the target population of this review was female graduate students.

## 5. Conclusions

Most studies in this review reported limited knowledge of breast cancer (e.g., risk factors, signs, and symptoms) and low uptake of preventive practices among graduate students. Even among students who demonstrated adequate knowledge regarding symptoms of breast cancer risk factors (e.g., family history, red meat consumption, alcohol consumption, smoking and sedentary lifestyle), had good knowledge about BSE, mammogram and CBE, and correctly identified the right age to start those prevention practices, most of the students did not practice prevention measures. BSE is an accessible screening method that can be performed at home. However, the low levels of BSE among students highlights the urgent need to promote BSE and other preventive measures among students. To address this gap and promote preventive behaviors, universities should implement evidence-based, theory-informed interventions, such as incorporating breast cancer education, BSE training, and opt-in digital reminders into campus health and wellness programs.

## Figures and Tables

**Table 1:** Study characteristics of included studies (*n* = 16).

Study ID	Title	Location	Aim of Study	Knowledge Prevalence	Practice Prevalence
Das et al. (2022) [34]	Knowledge and awareness of breast cancer (BC) and breast self-examination (BSE) among college-going female students in Delhi-NCR: a cross-sectional study.	India	To determine the extent of knowledge and awareness of breast cancer, its risk factors, early signs and symptoms, and BSE.	Prevalence was not reported, and there were no significant differences in the levels of education and knowledge.	Only 29.8% of graduate students had ever practiced BSE and age was significantly associated with BSE practice (*p* < 0.001).
Deeb et al. (2024) [35]	Nutrition knowledge, attitudes, and lifestyle practices that may lead to breast cancer risk reduction among female university students in Lebanon.	Lebanon	To assess nutrition knowledge, attitudes, and lifestyle practices (KAP) that may lead to BC risk reduction among female university students in Lebanon.	The study found that knowledge score was significantly associated with BSE practice (adjusted OR: 1.48, 95% CI: 1.29–1.70).	Almost 97% of the graduate students had poor practice, and only 3% had good prevention practices for breast cancer.
Haque et al. (2016) [36]	Cognizance and utilization of breast cancer screening among the health professional female students and staff of the University Kuala Lumpur, Royal College of Medicine Perak, Malaysia.	Malaysia	This study aimed to determine the relationship between sociodemographic factors and knowledge, attitude, and perception on BC screening.	The graduate medical students’ knowledge score was 62, which was higher than the undergraduates.	No separate prevalence for graduate students, but no significant relationship between any of the sociodemographic factors and BSE practice among graduate students.
Hussain et al. (2022) [37]	A national survey to assess breast cancer awareness among female university students in Pakistan	Pakistan	To assess the awareness of female university students about breast cancer’s risk factors, signs and symptoms, and breast cancer exam.	Insufficient knowledge of breast cancer risk factors (52.6%), signs and symptoms (69.45%).	Not measured.
Ishtiak et al. (2022) [38]	Knowledge, practice, and associated factors of breast self-examination among female university students of Bangladesh.	Bangladesh	To explore knowledge, practice, and associated factors of BSE among female university students of Bangladesh.	83.3% of the master’s students had a low total knowledge score, while 100% of the PhD students had a low total knowledge score.	Each additional year of education was associated with a 1.42 times higher chance of having knowledge about BSE (95% CI: 1.02–1.97, *p* = 0.036).
Mohammed et al. (2023) [39]	Knowledge, attitudes, and practices related to breast cancer self-examination among medical students at the University of Khartoum, Sudan.	Sudan	To assess the knowledge, attitudes, and practices of Sudanese medical students regarding breast self-examination (BSE).	High awareness of some breast cancer risk factors (e.g., aging, family history) but poor knowledge of others (e.g., early menarche).	Three-quarters (*n* = 222; 75.3%) of the 295 respondents in this study practiced BSE because they do not want to be diagnosed with BC.
Oudsema (2020) [40]	Screening mammography: Guidelines versus clinical practice.	United States	To understand the medical students’ and practitioners’ comprehension of breast cancer screening guidelines and the existing literature on breast cancer screening.	Good understanding among medical graduates.	Not measured.
Pirzadeh (2018) [41]	Application of the health belief model in breast self-examination by Iranian female university students.	Iran	To apply the Health Belief Model (HBM) in breast self-examination among female university students in Iran.	-	Results indicated significant associations between the education level and practice of BSE (*p* = 0.031).
Prachishre et al. (2024) [42]	Awareness and knowledge about breast cancer and breast self-examination among female students: A hospital based study.	India	To assess the awareness and knowledge about breast cancer and BSE among female students from SLN Medical College and a nearby women’s college.	Only 23.8% of graduate students correctly identified family history as a risk factor.	Not measured.
Samah et al. (2014) [43]	Relationship between body image and breast self-examination intentions and behavior among female university students in Malaysia.	Malaysia	To examine the relationship between body image satisfaction and both breast self-examination (BSE) intentions and behaviors.	Only 36.8% correctly identified the symptoms of BC.	54.5% never performed BSE in the past year, and 25.2% intended to perform BSE monthly in the next year.
Samah et al. (2016) [44]	Insufficient knowledge of breast cancer risk factors among Malaysian female university students.	Malaysia	This study aimed to evaluate breast cancer literacy among young female university students in the Klang Valley and Selangor, Malaysia.	The proportion of individuals with knowledge scores greater than the 50th percentile regarding breast cancer was 43.9%.	Graduate students (master’s and PhD) had a higher rate of performing BSE compared to undergraduates.
Sarker et al. (2022) [45]	Effectiveness of educational intervention on breast cancer knowledge and breast self-examination among female university students in Bangladesh: a pre-post quasi-experimental study.	Bangladesh	To assess the effect of an educational intervention program on breast knowledge and the practice of breast self-examination among young female students at a university in Bangladesh.	36% know how to perform BSE, the majority (63%) of the students do not know how to perform BSE, and 60.62% are aware of breast cancer symptoms.	The percentage of students performing BSE increased from 21.3% (pre-test) to 33.8% (post-test).
Sarker et al. (2022) [46]	Knowledge of breast cancer and breast self-examination practices and its barriers among university female students in Bangladesh: Findings from a cross-sectional study.	Bangladesh	To assess the knowledge of symptoms, risk factors, treatment modalities, and screening methods of breast cancer among female students and to examine the practice of BSE and the factors that hinder the practice of BSE.	55% of the graduate students had a poor level of knowledge, and 45% of the graduate students had a good level of knowledge.	Only one in five students (21%) have ever practiced breast self-examination.
Satapathy et al. (2022) [47]	Breast self-examination ractice among medical postgraduate female students of Southern Odisha: A cross-sectional study.	India	To assess the knowledge, attitude, and practice of postgraduates on BSE.	Pre-test mean scores: symptoms (2.99), risk factors (3.35), treatment (1.79), prevention (3.82), screening (1.82), BSE process (1.57). Post-test mean scores: symptoms (6.35), risk factors (7.56), treatment (4.63), prevention (7.14), screening (3.98), BSE process (3.94). All significant *p* < 0.001.	75.61% practiced BSE and only 23.17% practiced BSE monthly.
Sedrak et al. (2016) [48]	Cancer screening knowledge and attitudes of under and post-graduate students at Kasr Al Ainy School of Medicine, Cairo University, Egypt.	Egypt	To assess the level of knowledge concerning cancer screening among medical students, house officers (interns), and residents, and to explore their attitudes toward cancer screening practices.	A total of 96.6% of the respondents had heard about BSE before, while 99.3% were aware that BSE should be performed monthly.	Not measured.
Zhang et al. (2021) [49]	Relationship between demographic factors, health education, breast cancer-related knowledge, attitudes, and breast self-examination behavior among Chinese female college students: A structural equation analysis.	China	This study aimed to create a structural equation model to evaluate the associations among demographic factors, health education, breast cancer-related knowledge, attitudes, and breast self-examination in Chinese female college students.	On average, about 36.41% of the participants rightly identified the different BC risk factors.	Prevalence of BSE among postgraduate students: 45.5% (5 out of 11). Educational level was significantly associated with BSE performance (*p* < 0.05).

**Table 2:** Knowledge of breast cancer risk factors, signs, and symptoms reported in studies (*n* = 16).

Study	Knowledge of BC Reported by Study Authors	Knowledge of BC Risk Factors (%)	Knowledge of BC Signs and Symptoms (%)	Knowledge of Breast Cancer Screening Methods (%)	How Knowledge Was Measured
Das et al. (2022) [34]	Poor	36.41% rightly identified different BC risk factors.	42.02% had knowledge of one or other BC signs and symptoms.	51.2% know about BSE.	No total “knowledge score” was computed per participant; knowledge levels were reported as % of participants who answered each item (risk factors, sign and symptoms and BSE) correctly. 1 = has knowledge; 0 = no knowledge/do not know.
Deeb et al. (2024) [35]	Poor	68.3% of students had poor knowledge regarding nutrition-related breast cancer (BC) risk factors.	Not assessed.	Not assessed	Knowledge score was measured with 18 questions on modifiable and non-modifiable risk BC risk factors (0–18), categorizing knowledge levels as good knowledge = score ≥ 14 out of 18 and poor knowledge score: <14.
Haque et al. (2016) [36]	Good	87.7% identified genetic factors as a risk factors, 54.2% cited smoking and 52.2% cited sedentary lifestyle.	Not assessed.	76.8% know about mammograms.	Total knowledge score, categorized as low, average and high; items measured were risk factors and screening methods. Exact scoring rubric is not provided. A structured questionnaire was used.
Hussain et al. (2022) [37]	Poor	23.8% correctly identified family history as a risk factor.	24.2% correctly identified breast lumps as a symptom.	24.5% of PG students correctly stated that breast examination by a doctor/midwife should start after age 20.	No specific cutoff score but concluded poor level based on percentages as knowledge level was assessed item by item (risk factors, signs and symptoms, breast cancer examination), not as a composite score or categorized level.
Ishitiak et al. (2022) [38]	Moderate	Not assessed.	Not assessed.	Proportion was not calculated but the average knowledge score was 7.41 ± 3.27 out of 15.	Scored knowledge from 12 specific questions related to BSE. Each correct answer = 1 point. Incorrect or “Don’t know” answers = 0 points with a total score of 15.
Mohammed et al. (2023) [39]	Satisfactory	Not assessed.	Not assessed.	96.6% had heard of BSE.	Knowledge score derived from correct response to questions about knowledge items specific to general awareness of BSE and responses were summarized using frequencies and percentages that reflect the general knowledge level of the participants, which the authors described as “satisfactory” in the discussion section.
Oudsema (2022) [40]	Poor	Not assessed.	Not assessed.	74.7% felt very to somewhat uncomfortable with their knowledge of breast cancer screening.	No cutoff score but measured the % of correct responses of knowledge items on screening and mammography.
Pirzadeh (2018) [41]	Poor	77.6% correctly identified aging as a risk factor.	Not assessed.	17.6% know the correct steps of BSE.	Knowledge was measured using a questionnaire with 12 items focused on risk factors for breast cancer, higher scores indicating good level.
Prachishree et al. (2024) [42]	Poor	Not assessed.	60.2% of participants reported awareness of breast cancer symptoms.	42.8% had heard of mammography.	A structured questionnaire was used including questions on symptoms and preventive methods. Percentages and frequencies are used to present responses to individual knowledge-related questions.
Samah et al. (2014) [43]	Not measured	Not assessed.	Not assessed.	Not assessed.	Not measured.
Samah et al. (2015) [44]	Satisfactory	Only 5 out of 18 risk factors were correctly identified by over 60% of participants.	89.3% identified a lump in the breast as symptoms.	Not assessed.	Knowledge was measured using questions related to BC risk factor and symptoms. Median score was 52.6, > median score, considered satisfactory.
Sarker et al. (2022a) [45]	Poor	33.5% correctly identified risk factors in pre-intervention and 75.6% correctly identified risk factors in post intervention.	33.5% correctly identified risk factors in pre-intervention and 75.6% correctly identified risk factors in post intervention.	33.5% correctly identified risk factors in pre-intervention and 75.6% correctly identified risk factors in post intervention.	Total score of 43, greater score indicated good knowledge, 43 questions (symptoms—8 items, risk factors—10 items, treatment—6 items, prevention—8 items, screening methods—5 items, and process of BSE—5 items).
Sarker et al. (2022b) [46]	Poor	33.5% correctly identified risk factors.	36.8% correctly identified risk factors.	36.4% correctly identified screening methods.	Total score of 43, greater score indicated good knowledge, 43 questions (symptoms—8 items, risk factors—10 items, treatment—6 items, prevention—8 items, screening methods—5 items, and process of BSE—5 items).
Satyapathy et al. (2022) [47]	Good	100% knew family history/inheritance is a risk factor.	96.3% recognized a painless, hard lump with an uneven edge as a sign.	100% knew about BSE, CBE and mammograms.	Total knowledge score was 40 and 20 questions items were on knowledge of BSE. (A score of “2” was used for correct responses, “1” for do not know, and “0” for incorrect response in assessment of knowledge.) Those who scored > 50th percentile were categorized as having good knowledge.
Sedrak et al. (2016) [48]	Poor	Not assessed.	Not assessed.	70.7% correctly identified mammography and clinical breast exam as recommended for ages 40–49.	Total knowledge score of 24, scoring > 10 was considered knowledgeable, knowledge (total 24 knowledge questions about screening method), correct response assigned a score = 1 and incorrect or not sure scored = 0.
Zhang et al. (2021) [49]	Poor	68% correctly identified high-fat diet as a risk factor.	34% correctly identified nipple discharge as a symptom.	Not assessed.	11-item knowledge questions related to breast cancer symptoms, risk factors, and prevention were assessed using true and false options; total score was 11. Higher score indicates a better knowledge level.

Abbreviations: BC: breast cancer; BSE: breast self-examination; CBE: clinical breast examination.

**Table 3:** Factors associated with knowledge and preventive practice of breast cancer among graduate students.

Study	Factors Associated with Knowledge		Factors Associated with Preventive Practices	
	Significant	Not Significant	Significant	Not Significant
Das et al. (2016) [34]	Age	Smoking status, alcohol consumption, red meat consumption.	Age, pain in breast.	Physical inactivity, gene mutations, large breasts, experiencing a lump in breast or underarm region.
Deeb et al. (2024) [35]	Major of study, GPA	Age, smoking status, perceived vulnerability to stress.	Major of the study (e.g., health-related), BMI.	Age, marital status, smoking status, perceived vulnerability to stress.
Haque et al. (2016) [36]	Age	Background history of BC, educational qualifications.	-	Education, family history of BC, marital status.
Hussain et al. (2022) [37]	Level of education (e.g., undergraduate, graduate)	Marital status, major discipline, residence.	-	-
Ishitiak et al. (2022) [38]	Age, university type, education level (e.g., undergraduate, graduate)	Marital status, residence, father’s education, mother’s education, monthly family income.	Age, education (years), residence, fathers’ education, number of family members and knowledge score.	Marital status, university type, current residence, monthly family income.
Pirzadeh et al. (2018) [41]	-	-	Marital status and education level, perceived severity, self-efficacy.	Age, major.
Samah et al. (2014) [43]	-	-	Educational levels, income and age.	Race.
Samah et al. (2015) [44]	-	-	Education levels, ethnicity, and age.	-
Sarker et al. (2022) [46]	Age, education level (undergraduate vs. master’s), having family members with breast cancer.	Marital status, source of information.	Knowledge score, having family members with breast cancer.	Age, years of education, marital status, source of information.
Satyapathy et al. (2022) [47]	-	-	Knowledge on frequency of BSE.	Family history of breast cancer.
Sedrak et al. (2016) [48]	Years of enrollment in medical school.	Exam evaluation score at medical school, gender, age, lack of training about cancer screening.	-	-
Zhang et al. (2021) [49]	Academic level (e.g., undergraduate, graduate), age.		Knowledge of BSE, attitudes, health education, university type, major of study, birthplace, education (years), family history of BC, history of benign breast disease.	Ethnicity, mother’s education.

Abbreviations: BC: breast cancer; BMI: body mass index; BSE: breast self-examination; GPA: grade point average. Notes: Knowledge was measured using different items across the included studies (see Table 2).

## Data Availability

No new data were created or analyzed in this study. Data sharing is not applicable to this article.

## Supplementary Materials

The following supporting information can be downloaded at: https://www.mdpi.com/article/10.3390/cancers18071147/s1, Figure S1: Map of study locations, which included Malaysia (*n* = 3), Bangladesh (*n* = 3), India (*n* = 3), United States (*n* = 1), Lebanon (*n* = 1), Iran (*n* = 1), China (*n* = 1), Sudan (*n* = 1), Egypt (*n* = 1), and Pakistan (*n* = 1); Table S1. Search Strings Used for Databases; Table S2: Codebook for Data Extraction Variables; Table S3: Preferred Reporting Items for Systematic reviews and Meta-Analyses extension for Scoping Reviews (PRISMA-ScR) Checklist.

## Abbreviations

The following abbreviations are used in this manuscript:

BSE	Breast Self-Examination
BC	Breast Cancer
CBE	Clinical Breast Examination
CDC	Centers for Disease Control and Prevention
JBI	The Joanna Briggs Institute
PCC	Population, Concept, Context
