# Supplementary material for: Breast Cancer Knowledge and Preventive Practice Among Graduate Students: A Scoping Review
Source: Cancers (Basel). 2026 Apr 2;18(7):1147. doi: 10.3390/cancers18071147 (PMC13072285; doi:10.3390/cancers18071147)
Supplement: Supplementary file 1 [file cancers-18-01147-s001.zip › cancers-4222146-supplementary.pdf]

cancers

Supplementary Materials

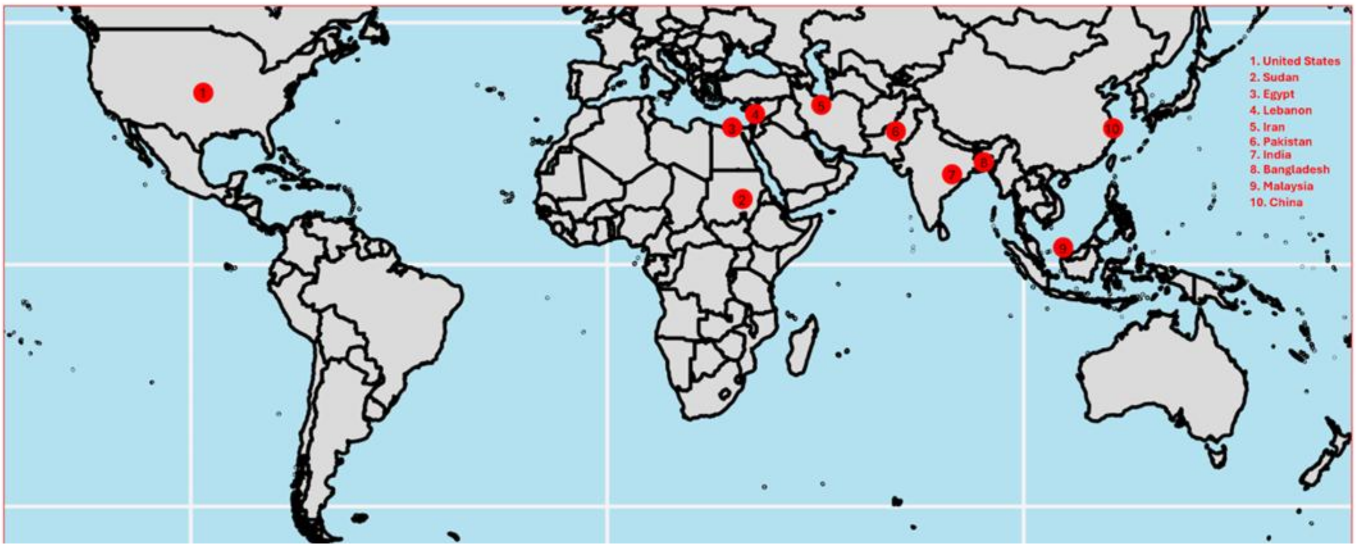

Figure S1. Map of study locations, which included Malaysia (*n* = 3), Bangladesh (*n* = 3), India (*n* = 3), United States (*n* = 1), Lebanon (*n* = 1), Iran (*n* = 1), China (*n* = 1), Sudan (*n* = 1), Egypt (*n* = 1), and Pakistan (*n* = 1).

Table S1. Search Strings Used for Databases.

Databases	Search Terms	Results
PubMed	(((("Breast neoplasms"[Mesh] OR breast neoplasm*[tiab] OR breast cancer*[tiab] OR breast carcinoma [tiab] OR breast tumor [tiab])) AND (("early detection of cancer" [MeSH] OR early detect* [tiab] OR breast self examination [MeSH] OR "mass screening" [MeSH] OR screening [tiab] OR "cancer screening" [tiab] OR "screening practices" [tiab] OR practices [tiab]) OR (prevention [tiab]))) AND ((knowledge OR awareness))) AND (("university students" OR "graduate students" OR "college student" OR "medical students" OR students, health occupations [MeSH]))	76
CINAHL	-	20
APA PsycINFO	-	4
EMBASE	-	193

Table S2. Codebook for Data Extraction Variables.

Domain	Component	Definition
Study Identification	Study ID	Unique identifier assigned to each included study for reference.
Study Identification	Title	Full title of the study as published.
Study Identification	First Author	Name of the first author listed on the study.
Study Identification	Year of Publication	The study was published in a peer-reviewed journal.
Study Identification	Country	Country where the study was conducted.
Study Identification	Journal	Name of the journal in which the study appeared.
Study Characteristics	Study Type	Design of the study (e.g., cross-sectional, qualitative, mixed methods).

 https://doi.org/10.3390/cancers18071147

Study Characteristics	Sample Size	Total number of participants included in the study.
Study Characteristics	Target Population	Description of the participants (e.g., female graduate students).
Study Characteristics	Setting	Physical or institutional setting of the study (e.g., university campus).
Overall Breast Cancer Knowledge	Knowledge of BC Reported by Authors	Whether the authors reported that participants had knowledge of breast cancer in general. The overall knowledge of breast cancer was reported by authors using validated/unvalidated questionnaire using different knowledge score and proportions.
Knowledge of risk factors	Proportion with Knowledge of Risk Factors	Percentage or proportion of participants who had knowledge of breast cancer risk factors.
Knowledge of signs & Symptoms	Proportion with Knowledge of Signs & Symptoms	Percentage or proportion of participants who were aware of breast cancer signs and symptoms.
Knowledge of Breast cancer screening methods	Proportion with Knowledge of Screening Methods	Percentage or proportion of participants who were aware of breast cancer screening methods (BSE, CBE, mammogram).
Preventive Practices	Proportion of students participating in breast cancer screening methods (BSE, CBE & Mammogram)	Percentages or proportion of students reported undergoing Clinical Breast Examination (CBE), Mammogram and BSE.

Table S3. Preferred Reporting Items for Systematic reviews and Meta-Analyses extension for Scoping Reviews (PRISMA-ScR) Checklist.

SECTION	ITEM	PRISMA-ScR CHECKLIST ITEM	REPORTED ON PAGE #
		**TITLE**	
Title	1	Identify the report as a scoping review.	1
		**ABSTRACT**	
Structured summary	2	Provide a structured summary that includes (as applicable): background, objectives, eligibility criteria, sources of evidence, charting methods, results, and conclusions that relate to the review questions and objectives.	1
		**INTRODUCTION**	
Rationale	3	Describe the rationale for the review in the context of what is already known. Explain why the review questions/objectives lend themselves to a scoping review approach.	3
Objectives	4	Provide an explicit statement of the questions and objectives being addressed with reference to their key elements (e.g., population or participants, concepts, and context) or other relevant key elements used to conceptualize the review questions and/or objectives.	4
		**METHODS**	
Protocol and registration	5	Indicate whether a review protocol exists; state if and where it can be accessed (e.g., a Web address); and if available, provide registration information, including the registration number.	N/A
Eligibility criteria	6	Specify characteristics of the sources of evidence used as eligibility criteria (e.g., years considered, language, and publication status), and provide a rationale.	5
Information sources*	7	Describe all information sources in the search (e.g., databases with dates of coverage and contact with authors to identify additional sources), as well as the date the most recent search was executed.	4

SECTION	ITEM	PRISMA-ScR CHECKLIST ITEM	REPORTED ON PAGE #
Search	8	Present the full electronic search strategy for at least 1 database, including any limits used, such that it could be repeated.	4
Selection of sources of evidence†	9	State the process for selecting sources of evidence (i.e., screening and eligibility) included in the scoping review.	5-6
Data charting process‡	10	Describe the methods of charting data from the included sources of evidence (e.g., calibrated forms or forms that have been tested by the team before their use, and whether data charting was done independently or in duplicate) and any processes for obtaining and confirming data from investigators.	5-6
Data items	11	List and define all variables for which data were sought and any assumptions and simplifications made.	6-9
Critical appraisal of individual sources of evidence§	12	If done, provide a rationale for conducting a critical appraisal of included sources of evidence; describe the methods used and how this information was used in any data synthesis (if appropriate).	N/A
Synthesis of results	13	Describe the methods of handling and summarizing the data that were charted.	6
**RESULTS**			
Selection of sources of evidence	14	Give numbers of sources of evidence screened, assessed for eligibility, and included in the review, with reasons for exclusions at each stage, ideally using a flow diagram.	5
Characteristics of sources of evidence	15	For each source of evidence, present characteristics for which data were charted and provide the citations.	6
Critical appraisal within sources of evidence	16	If done, present data on critical appraisal of included sources of evidence (see item 12).	N/A
Results of individual sources of evidence	17	For each included source of evidence, present the relevant data that were charted that relate to the review questions and objectives.	8-10
Synthesis of results	18	Summarize and/or present the charting results as they relate to the review questions and objectives.	8-13
**DISCUSSION**			
Summary of evidence	19	Summarize the main results (including an overview of concepts, themes, and types of evidence available), link to the review questions and objectives, and consider the relevance to key groups.	14-16
Limitations	20	Discuss the limitations of the scoping review process.	16
Conclusions	21	Provide a general interpretation of the results with respect to the review questions and objectives, as well as potential implications and/or next steps.	17
**FUNDING**			
Funding	22	Describe sources of funding for the included sources of evidence, as well as sources of funding for the scoping review. Describe the role of the funders of the scoping review.	17

JBI = Joanna Briggs Institute; PRISMA-ScR = Preferred Reporting Items for Systematic reviews and Meta-Analyses extension for Scoping Reviews. * Where *sources of evidence* (see second footnote) are compiled from, such as bibliographic databases, social media platforms, and Web sites. † A more inclusive/heterogeneous term used to account for the different types of evidence or data sources (e.g., quantitative and/or qualitative research, expert opinion, and policy documents) that may be eligible in a scoping review as opposed to only studies. This is not to be confused with *information sources* (see first footnote). ‡ The frameworks by Arksey and O'Malley (6) and Levac and colleagues (7) and the JBI guidance (4, 5) refer to the process of data extraction in a scoping review as data charting. § The process of systematically examining research evidence to assess its validity, results, and relevance before using it to inform a decision. This term is used for items 12 and 19 instead of

"risk of bias" (which is more applicable to systematic reviews of interventions) to include and acknowledge the various sources of evidence that may be used in a scoping review (e.g., quantitative and/or qualitative research, expert opinion, and policy document).
